# Statistical analysis of the effect of socio-political factors on individual life satisfaction

**DOI:** 10.1038/s41598-024-70067-5

**Published:** 2024-08-24

**Authors:** Alaa Itani, Isra Hasan, Lolya Younes, Ayman Alzaatreh

**Affiliations:** 1 Department of Psychology, American University of Sharjah, Sharjah, UAE; 2 Department of Computer Science and Engineering, American University of Sharjah, Sharjah, UAE; 3 Department of Mathematics and Statistics, American University of Sharjah, Sharjah, UAE

**Keywords:** Life satisfaction, Socio-political, World values survey, Confirmatory factor analysis, Structural equation modeling, Psychology and behaviour, Statistics

## Abstract

Life satisfaction refers to an individual’s cognitive evaluation of the quality of their life. The aim of the present study is to develop the current understanding of how perceived corruption, attitudes toward migration, perceived security, and strength of national identity influence life satisfaction. Additionally, the study examines how demographic variables of relationship status, social class, sex, religious affiliation, and country impact life satisfaction in the provided cultural context. Ordinal logistic regression analysis, Confirmatory Factor Analysis, and Structural Equation Modeling are used to analyze data from the World Values Survey. Findings from the analyses indicate that perceived corruption, perceived security, and strength of national identity have a significant impact on life satisfaction, whereas migration has an indirect effect on life satisfaction through perceived security. The present research can develop our current understanding of life satisfaction from a socio-political perspective.

## Introduction

An individual’s life satisfaction refers to the cognitive and affective evaluation of the quality of their life^1^. Researchers often use life satisfaction as an indicator of subjective well-being, considering life satisfaction as a cognitive evaluation and happiness as an affective evaluation of positive psychological health^2^. According to Diener et al.^2^, individuals across 41 nations rated both life satisfaction and happiness as highly important, reflecting the value individuals place on life satisfaction. Based on self-report measures and positive outcomes across psychological, physiological, and organizational domains, research has also demonstrated the importance of life satisfaction.

Psychologically, lower levels of life satisfaction are associated with outcomes such as distress, depression, and anxiety. Meanwhile, higher levels of life satisfaction are associated with greater resilience in the face of adversities^3^. Physiologically, elevated life satisfaction is linked to reduced sleep complaints and lower rates of cardiovascular and overall mortality^4,5^. Organizationally, life satisfaction contributes to lower levels of burnout, enhanced job performance, and decreased intentions to quit^6–8^.

Governments are increasingly incorporating happiness and life satisfaction into public policies and national agendas. Gross National Happiness (GNH) was developed in Bhutan as an indicator of population happiness to inform policies, develop programs, and track national progress, a move praised by the United Nations^9^. Similarly, the European Union’s project, Bringing Alternative Indicators into Policy (BRAINPOoL), explored indicators that can be used for policy-making, including indicators of happiness and well-being^9,10^. Currently, the United Kingdom is emphasizing happiness in policy-making and investigating methods to measure happiness among its citizens^9^. Given the trend toward using happiness as an affective measure of personal and national success, it is critical to consider the factors that influence life satisfaction, given its relation to happiness and well-being. Here, we recognize and assess the dimensions associated with the socio-political domain to evaluate the impact of beliefs and attitudes on life satisfaction.

To explore these dimensions, we turn to the world values survey (WVS), which collects data from over 80 countries about various indicators, including life satisfaction and other socio-political factors relevant to the current study^11^. This study uses the WVS to assess the relationship between life satisfaction and several socio-political factors at a micro-level of analysis, including corruption, attitudes toward migration, security, and national identity. Following measurement validation, we argue that these four socio-political factors influence life satisfaction and assess how our hypothesized conceptual model fits the collected data.

Given the importance of life satisfaction to people around the globe as well as its various positive outcomes, it is important to understand the interlinked factors that influence life satisfaction, especially in socio-political spheres where there is a divide between micro and macro levels of research^12^. Specifically, the current study aims to develop an understanding of how socio-political beliefs, values, and perceptions can influence life satisfaction in the shifting socio-political landscape shaped by COVID-19 using data collected in 2021. We contextualize individual satisfaction within the broader socio-political scene from a micro-level, ultimately striving to promote life satisfaction through socio-political policies and initiatives.

First, we use confirmatory factor analysis (CFA) to determine whether items on the WVS reflect the factors they are intended to measure. Second, we used structural equation modeling (SEM) to assess the nature and strength of the direct relationships between factors (corruption, migration, security, and national identity) and life satisfaction. Additionally, we investigate how individual-level variables such as country, sex, relationship status, socioeconomic status, and religious denomination impact life satisfaction in specific cultural contexts. We then use SEM controlled by country to investigate whether the path structure would maintain its validity across countries, using the findings of the multi-group analysis to develop an updated framework for life satisfaction and its related factors. The goal is to inform strategies at the macro-level of social institutions, political systems, and organizational structures to enhance life satisfaction at the micro-level of individual perceptions, attitudes, and beliefs.

## Literature review

Many researchers have investigated individual and relational factors that contribute to life satisfaction. Several authors, for example, have concluded that individual factors such as self-esteem, optimism, extraversion, and personal control all contribute to life satisfaction^13,14^. When it comes to relational factors, relationships with people (e.g. social support) and relationships with God (e.g. religion/spirituality) significantly influence life satisfaction^15,16^. According to Bohnke^17^, employment, job satisfaction, and health are also associated with more life satisfaction. Given the various factors that influence life satisfaction as well as the many positive psychological, physiological, and organizational outcomes of life satisfaction, it is increasingly important to analyze life satisfaction in a post-pandemic world where anxiety, burnout, poor sleep quality, depressive symptoms, cognitive impairments, and poor quality of life have been on the rise^18^.

### Perceived corruption

Corruption involves abusing and exploiting public resources for the purposes of personal interest and private gain^19^. Corruption threatens democratic outputs and policies^20^, reducing economic investments, exacerbating economic inequality, and undermining the quality of institutions in a state^21^. Research suggests that individuals residing in nations characterized by a high prevalence of corruption tend to experience lower levels of life satisfaction compared to those residing in countries where corruption is less prevalent^22^.

Studies indicate that the perception of governmental corruption is associated with a negative impact on subjective well-being^23^. Perceived corruption erodes the relationship between individuals and the state. Importantly, individuals who perceive more corruption tend to be less trusting of their governments compared to those who perceive less corruption, especially when they cannot punish corrupt individuals and hold them accountable^21,24^. With a weak relationship to the state, individuals who perceive more corruption report lower levels of satisfaction with life and subjective well-being than individuals who perceive less corruption^25^. Given the adverse effects of individuals' perceptions of corruption on their subjective well-being, we investigate whether beliefs about corruption influence life satisfaction in the current geopolitical context.

#### H_1_

Perceived corruption has a significant impact on individuals’ satisfaction

### Attitudes toward migration

With the rise of globalization, the number of migrants has increased, reaching 272 million migrants in 2019 worldwide, with over 750 million individuals expressing a desire to migrate if they could^26,27^. While there are contested definitions of what makes someone a migrant, a typical migrant is someone who moves to a new country for a period of time long enough for the new country to become their usual residence^28^. There are both economic and non-economic determinants of attitudes toward migration. Economically, anti-immigration attitudes arise when the skill set of citizens (natives) is similar to that of immigrants^29^. When the skill sets are too similar, competition arises in the labor market, and anti-immigrant attitudes increase and become more salient than when the skill sets of citizens and immigrants are different and no economic competition ensues^30^. Apart from economic considerations, racial, ethnic, and cultural considerations are also important^31^. For example, Dustmann and Preston^32^ found that racial and cultural prejudice are important contributors to attitudes toward migration.

Both economic and non-economic factors play a significant role in attitudes toward migration. Namely, negative attitudes toward migration are often shaped by a feeling of threat, such as a threat to one’s employment, safety and order, or cultural values and practices^33,34^. Perceived threats, such as ones related to immigrants, can undermine life satisfaction. For example, perceived financial threats diminish life satisfaction^35^, and attitudes toward migration negatively impact life satisfaction among Europeans^36^. Here, we use different threats to determine attitudes toward migration, and then test whether endorsed attitudes predict life satisfaction.

#### H_2_

Attitudes toward migration have a significant impact on individuals’ satisfaction

### Perceived security

Looking at how economic competition and racial intolerance influence migration attitudes, other factors influence perceived security, which is another socio-political factor that can develop our understanding of life satisfaction. Human security is characterized by safety and protection from harmful, chronic, or unanticipated threats (e.g. disease)^37^. While feelings of safety and protection from threats might not reflect reality, they significantly contribute to one's overall sense of perceived security. Notably, perceived security is negatively linked to the fear of war, fear of crime, and safety anxiety. For example, research indicates that individuals who value security reported greater fear of war^38^. Such fears can have a negative impact on both the individual and society. On the individual, evidence suggests that safety anxiety can restrict freedom of mobility out of fear of being a target of harm, inducing a state of chronic hypervigilance^39^.

At the societal level, the fear of crime increases economic expenditure, violent attitudes, and crime; fear of crime also reduces trust in authorities and willingness to help^40^. Recognizing the adverse effects of diminished perceived security on both the individual and society, it becomes clear that perceived security is an important contributor to subjective well-being. Studies have demonstrated that participants who reported feeling unsafe exhibited lower levels of life satisfaction^41^. In another study, fear of crime was negatively associated with life satisfaction^42^. Seeing how important feeling safe is to one’s well-being, we include perceived security as a factor in this analysis to explore its relationship and impact on life satisfaction, taking into consideration its interaction with other socio-political factors that constitute threats, such as migration. Although previous research has studied security, this study reexamines the impact of perceived security on life satisfaction within the distinct socio-political contexts of selected countries, avoiding the generalization of existing findings to specific contexts.

#### H_3_

Perceived security has a significant impact on individuals’ satisfaction

### National identity

National identity, while abstract and multidimensional, can be an important aspect of one’s social identity^43^. National identity encompasses the territorial distinctiveness of cultural groups, shared origin myths and historical memories, a unified mass culture, territorial division of labor and resource ownership, and a common system of legal rights and duties^44^. National identity relates to how individuals associate with characteristics unique to their nation-state, including religion, history, customs, and social institutions^45^.

Evidence suggests that a stronger sense of national identity correlates with positive psychological outcomes. For example, Khan and colleagues^46^ found that a strong association with one’s nation predicted improved health outcomes and lower levels of anxiety. Researchers have also found evidence between national identity and self-esteem, post-traumatic growth, interpersonal trust, and subjective well-being^47^.

A stronger national identity is also linked to higher levels of life satisfaction^48^. Across ethnic and cultural groups, there is a positive relationship between how strongly one identifies with their nation and their satisfaction with life^48–50^. When individuals identify with the nation, they develop meaning systems that stem from the nation's values, beliefs, and attitudes^43^. National identity provides individuals with a sense of belonging, connection to a greater purpose, and meaning in life, thereby enhancing life satisfaction^43^.

However, the relationship between life satisfaction and national identity is not culturally robust and is an area of further research. For example, Jordanov et al.^50^ found that national identity was associated with life satisfaction among Romanian youth, but this relationship did not extend to Bulgarians. Similarly, national identity did not significantly predict subjective well-being among Qataris following a period of national adversity^51^. Here, we look at global trends of national identity across 50 countries, providing cross-cultural evidence of the link between a stronger identification with the nation and an individual’s life satisfaction.

#### H_4_

The strength of national identity has a significant impact on individuals’ satisfaction

### Demographic variables

Although socio-political factors are important for understanding life satisfaction, demographic factors can also shape our experiences and emotions, including life satisfaction. However, research on the impact of demographic variables on life satisfaction provides mixed results and presents conflicting evidence on their role in influencing life satisfaction. Therefore, it is essential to investigate how demographic variables of sex, relationship status, social class, religious denomination, and country relate to life satisfaction in the context of the present study. Indeed, it is important not to make assumptions about generalizability, as boundary conditions such as culture or environment can vary across ecological contexts.

#### Relationship status

Even though being in a relationship fulfills some of our most essential social needs, the number of single individuals has been on the rise as people find it more difficult to date in the present day^52^. In 2019, 38% of U.S. adults reported being single^53^. Specifically, single individuals who are not looking for a relationship report that they enjoyed being single or had more important life priorities^54^. However, being single is also associated with worse educational and economic outcomes than being in a relationship^53^. Additionally, empirical evidence indicates that individuals in relationships tend to report higher levels of life satisfaction compared to single individuals. For example, Adamczyk and Segrin^55^ found that individuals who were single reported lower levels of life satisfaction, social support from a significant other, and loneliness.

However, other studies present findings that life stressors and changes can impact relationships, as stressors are associated with reduced desire for emotional and physical closeness^56^. In addition to the stresses of everyday life, social media and COVID-19 have had significant impacts on the nature and perceptions of dating, relationships, and social support. For example, social media use is associated with conflict in relationships^57^. Additionally, while COVID-19 presented new opportunities for couples to spend time together and promote intimacy in the relationship, it also presented new interpersonal challenges, creating new sources of distress and dysfunction in relationships^58^. As a source of both opportunities and challenges, the complexity of relationships is highlighted in the literature. For example, Adamczyk and Segrin^55^ found no direct effect of relationship status on life satisfaction. Instead, the indirect effect of relationship status on life satisfaction was through social support and loneliness. As societal expectations of marriage and relationships are changing in a world dominated by daily stressors, dating apps, and social media, alternative paths of social support present themselves, providing alternatives for meeting social needs and altering the impact of relationship status on individual well-being. Importantly, the current research looks at whether the effect of relationship status on life satisfaction in the contexts of interest is consistent with the research, especially given the dynamic and changing nature of the landscape of relationships.

#### Social class

**Figure 1:**
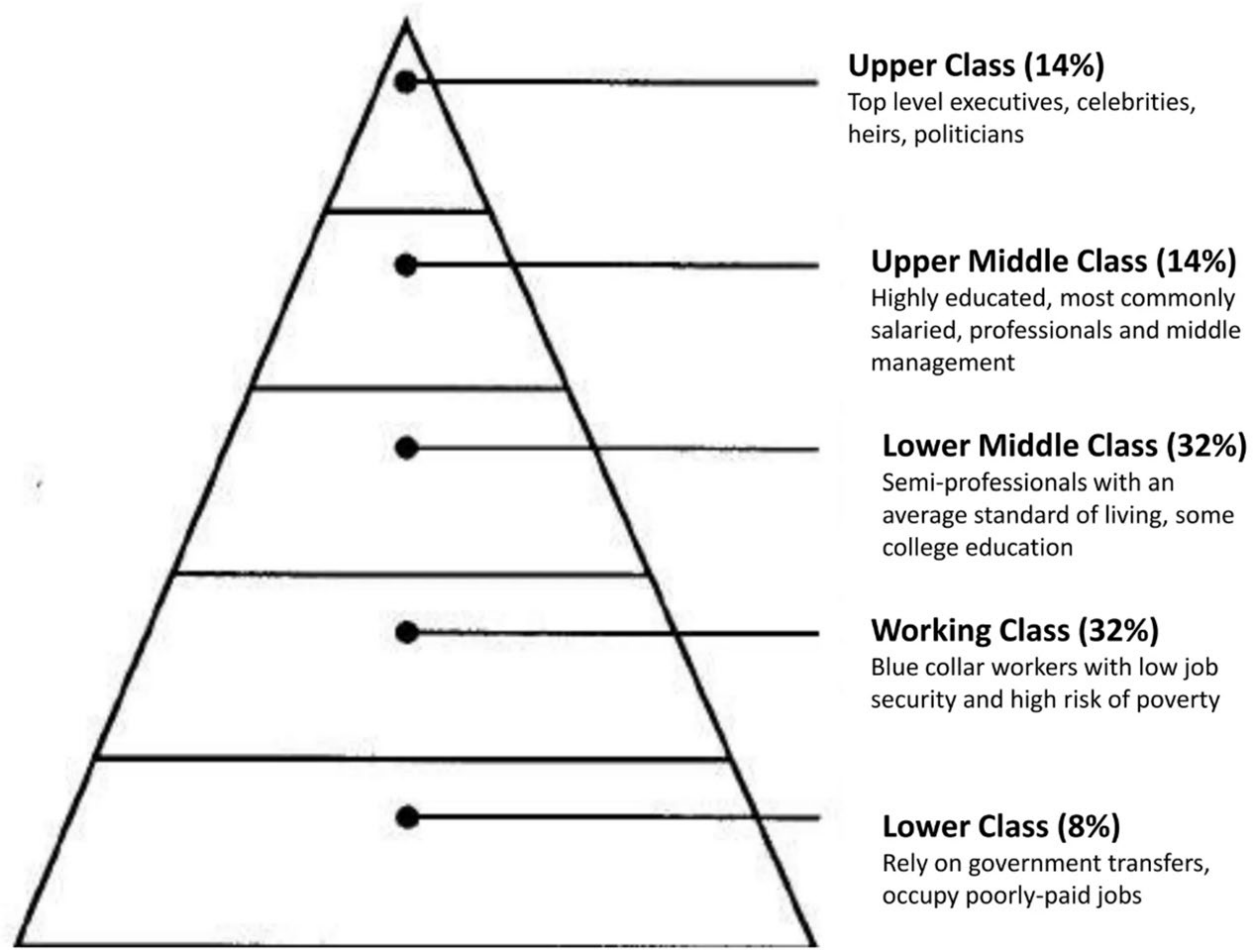
Social class pyramid. Upper class, upper middle class, lower middle class, working class, and lower class descriptions and proportions in a typical capitalistic society, such as the U.S.^64^.

Social class and socioeconomic status can significantly shape our lives, both through economic and social means. At the national level, countries with higher incomes have greater life satisfaction than low-income countries^59^. Specifically, socioeconomic status can influence life satisfaction by satisfying the needs of individuals^60^. It is unsurprising, then, that there is a positive relationship between socioeconomic status and life satisfaction^61^. Research on data from 41 countries found that individuals from more affluent families reported higher levels of life satisfaction compared to those from less affluent families^62^. Here, we define social class as encompassing social and economic factors that categorize individuals in a society. For example, social class can be determined by access to financial resources, educational opportunities, occupational prestige, and social standing^63^. Specifically, we categorize social class into upper class, upper middle class, lower middle class, working class, and lower class based on respondents' self-categorization^64^ (see Fig. 1).

Importantly, cross-national research on life satisfaction has often excluded countries investigated in this research. For example, Armenia is sometimes excluded from analyses in satisfaction research due to outlying scores^62,65^. While national-level data is available for countries such as Venezuela, Kenya, Morocco, and the Maldives, their populations remain understudied. In particular, ecological factors in these countries might yield patterns of results that diverge from the literature and the traditional relation between socioeconomic status and life satisfaction^66^. For example, the sudden shift to a market in countries with a history of communism, such as Armenia, can cause periods of transitional stress, reducing life satisfaction^67^. Consequently, our research sought to determine whether the positive relation between socioeconomic status and life satisfaction would extend to these countries.

#### Sex

Differences in biological sex are often associated with differences in affect, cognition, and behavior^68^. For example, males are less prone to developing psychological problems, more likely to outperform females on motor performance and mental rotation tasks, and engage in more risk-taking behaviors than females^69,70^. Despite researchers commonly including sex in studies about life satisfaction, the relationship between life satisfaction and sex remains unclear^71^. While some studies report that males tend to be less satisfied than females, other studies have reported opposite findings^71^. Some research has found no differences in satisfaction between males and females^72^. Many researchers have noted that environmental factors such as culture, gender stereotypes, gender equality, learning experiences, and biosocial interactions all influence the current understanding of gender differences^70^. Taking account of such environmental influences, recent research has challenged some ideas about differences between sexes, and recent findings support that there are little to no sex differences in most domains^70^. Given the lack of clarity and consensus on the current influence of sex on life satisfaction, we sought to examine the relationship between the two.

#### Religious affiliation

Religion characterizes both individuals and societies. Okulicz-Kozaryn^73^ argued that, while social religiosity encompasses attending religious services, spending time with others at religious events, and belonging to religious organizations, individual religiosity relates to the belief in a higher being and the importance of religion. Literature on the relation between religiosity and life satisfaction presents conflicting evidence, with 80% of studies reporting a positive association, 13% of studies reporting no relationship, 7% finding mixed results, and one study reporting a negative correlation between the two^74^. Despite the majority of studies supporting the positive association between religion and life satisfaction, recent research has challenged the influence of religion on life satisfaction. For example, Lim and Putnam^75^ found that social religiosity facilitates social support and develops social networks, thereby indirectly enhancing life satisfaction. They found little evidence, however, that aspects of individual religiosity influence life satisfaction^75^.

Such findings on social religiosity align with other studies comparing religious affiliates to atheists and agnostics. For example, Hayward et al.^76^ found that religious affiliates had better psychological well-being, social support, and health behaviors than atheists and agnostics. Other studies find no differences in life satisfaction between religious affiliates and non-affiliates^77^. Not only is the research on the relationship between religion and life satisfaction unclear, but it is also contextual, with religious people reporting more satisfaction in religious than non-religious nations^73,78^. Atheists also had higher levels of well-being in countries with secular populations, indicating that social context is important for life satisfaction with respect to religion^77^. However, Uğur and Aydın^78^ found that most non-religious individuals did not feel pressured by societal expectations regarding religiosity. Overall, the conflicting evidence in the literature as well as the context-dependent nature of the relation between religion and life satisfaction make religious affiliation an important variable to examine in the present study.

#### Country

Across countries, life satisfaction can differ due to a variety of ecological factors. Chapman et al.^79^ highlighted the role of country or region in the way participants responded to surveys on life satisfaction. Even when variables are controlled, individuals report different levels of life satisfaction across socio-cultural environments, suggesting that the relationship between country and life satisfaction is more complex than it appears to be^80^. Specifically, cross-national research provides evidence that national indicators such as national wealth, environmental conditions, and human development are important when considering life satisfaction in a regional or national context^81^. Additionally, being satisfied with one’s country is positively correlated with their subjective well-being, suggesting a relation between national-level and individual-level factors^82^.

At the national level, economic factors such as labor market policies, energy affordability, and economic growth have been shown to influence life satisfaction^79,83^. However, national conditions related to security, health, equality, and politics are also associated with life satisfaction, suggesting that looking at the effects of regional context is complex and not adequately accounted for using economic indicators^79,84^. While economic factors might not completely account for life satisfaction, they still play an important role at the national level. For example, post-material values such as personal autonomy are more important for life satisfaction than material values such as income in affluent countries^85^. In the current research, we investigate data collected in five countries, including Armenia, Kenya, Morocco, Maldives, and Venezuela. In 2021, the gross domestic product (GDP) varied across these countries, with US$9808 per capita in the Maldives, US$4522 per capita in Armenia, US$3291 per capita in Morocco, US$2070 per capita in Venezuela, and US$1705 per capita in Kenya^86,87^. Research on the relationship between GDP and life satisfaction varies. While some evidence supports the positive relationship between GDP and life satisfaction^88^, Proto and Rustichini^89^ depict a more complex relationship between the two variables. Since life satisfaction significantly varies at the national level, we sought to determine the impact of one’s country on life satisfaction in the five aforementioned countries^81^.

In summary, while evidence indicates that socio-political factors are important for life satisfaction, there is little research providing information on how to cluster such factors that most influence life satisfaction, leaving a gap in the literature that we intend to address. Here, we examine the relationship between life satisfaction and perceived corruption, attitudes toward migration, perceived security, and strength of national identity. Additionally, because they are impacted by contextual factors, mixed results on the impact of individual-level variables such as sex, relationship satisfaction, social class, and religious denomination on life satisfaction leave another gap in the literature. Here, we investigate the role of these demographic variables on life satisfaction within the relevant cultural context. Our research, therefore, seeks to address these two gaps and promote life satisfaction.

## Methodology

In this study, the methodology involved several key steps:

We used data from the seventh wave of the World Values Survey (WVS) conducted in 2021. Data collection of the seventh wave, the most recent wave, began in 2017 and concluded in 2021 in collaboration with academic, research, and non-governmental organizations^11^. The survey in the seventh wave includes questions about self-reported social beliefs, attitudes, and values, including happiness and well-being, corruption, migration, religious values, and others. For its survey, the WVS gathered data through interviews with individuals aged 18 and above^11^. The data are freely available and can be accessed at https://www.worldvaluessurvey.org/wvs.jsp.

We began with cleaning the WVS dataset to include associated dimensions and their corresponding items, based on the categorization in the WVS and related literature^11,90^. We then used measurement validation to select items that measure each dimension. Missing data were removed, and full information maximum likelihood was used in the Structural Equation Modeling (SEM). Although the primary language of the master questionnaire is English, the language of instruction was translated based on the linguistic context of each region to enable speakers of different languages to respond.

Secondly, we conducted a confirmatory factor analysis (CFA) to measure the four socio-political dimensions (perceived corruption, attitudes toward migration, perceived security, and strength of national identity) using the validated items from the WVS and model validation using goodness of fit tests. Finally, we conducted SEM to evaluate the impact of the exogenous dimensions (perceived corruption, attitudes toward migration, perceived security, and national identity) on the endogenous dimension (life satisfaction).

### Survey design

Based on the previous literature review, we used questions from the WVS addressing demographic information, life satisfaction, and four factors related to socio-political beliefs, including perceived corruption, attitudes toward migration, perceived security, and strength of national identity. Items for each factor were selected based on the pre-existing classification by the World Values Survey (WVS), related literature, reliability analyses, and CFA analysis (refer to Sect. "Data analysis" for more details)^11,90^. In this section, we outline each measure, including its items and the scale it uses. The following section explains how items were selected for each factor. Details of selected items from the WVS, corresponding coded items, and a complete list of questions, are provided in Table S1.

#### Demographics

While the WVS includes many demographic variables, we focused on key demographic questions about sex, age, ethnicity, religious denomination, and socioeconomic status to contextualize our sample and understand the background of our responses. Additionally, we examined variables such as relationship status, employment status, and country. Data collected in 2021 overlapped with the COVID-19 pandemic, which greatly contributed to reshaping some demographic variables. Economically, the pandemic caused extensive unemployment, economic instability, and income fluctuations, leading to a significant gap between different social classes. Additionally, social distancing presented psychological challenges globally, significantly impacting emotional connections and mental health, as well as potentially straining relationships^91^.

**Figure 2:**
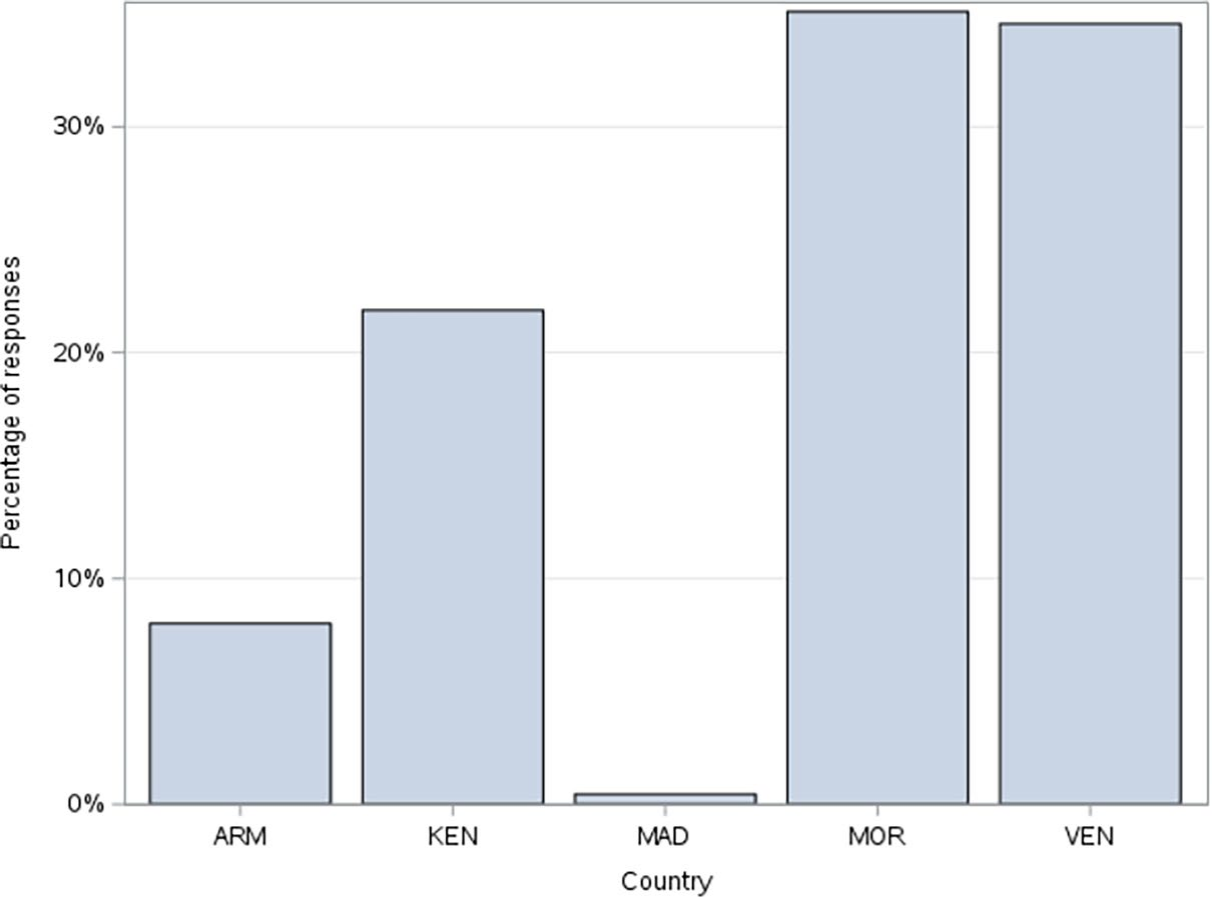
Bar chart showing the percentage of responses from each Country (2021). Data was collected from Armenia (ARM), Kenya (KEN), Morocco (MOR), Venezuela (VEN), and Maldives (MAD).

The data primarily included responses from five countries: Armenia, Kenya, Morocco, Maldives, and Venezuela (See Fig. 2). The final cleaned data consists of 3334 participants (1628 Males, 1706 Females) with a mean age of 36.67 (*SD* = 14.17). Participants were asked about their socioeconomic status (Upper Class = 1.7%, Upper Middle Class = 17.7%, Lower Middle Class = 42.7%, Working Class = 24.7%, Lower Class = 13.2%). For their ethnic groups, 35.14% of the participants were White, 11.84% were Black, 13.48% were South Asian Indian or Pakistani, 2.86% were East Asian Chinese or Japanese, 33.23% were Arab or Central Asian, and 3.45% reported that they identified with “other” ethnic groups. For their religious denomination, 27.9% were Roman Catholic, 17.27% were Protestant, 7.6% were Orthodox, 0.1% were Jew, 37.1% were Muslim, and 1.6% reported that they belonged to “other” religious denominations.

#### Life satisfaction

We used a single item as a measure of life satisfaction (See Table S1 for a full list of items). The question measured the extent to which participants were satisfied with their lives as a whole in the present. The life satisfaction question asks: All things considered, how satisfied are you with your life as a whole these days? The item uses a 10-point Likert-type scale ranging from “Completely dissatisfied” (1) to “Completely satisfied” (10). Higher scores on the ten items indicate higher levels of life satisfaction.

#### Corruption

Four items were selected C1, C2, C3, and C4, as a measure of perceived corruption (See Table S1 for a full list of items). These questions measured the extent to which participants perceived corruption among different social groups, including state authorities, business executives, local authorities, and civil service providers. The items use a 4-point Likert-type scale ranging from “none of them” (1) to “all of them” (4). Higher scores on the four items indicate higher levels of perceived corruption.

#### Migration

Four items were selected (M1, M2, M3, and M4) as a measure of attitudes toward migration (See Table S1 for a full list of items). These questions measured participants’ self-reported beliefs about the effects of immigration on the development of their country, including the effect of immigration on crime rates, risk of terrorism, unemployment, and social conflict. The items use a 3-point Likert-type scale ranging from “Agree” (2) to “Disagree” (0). Higher scores on the three items indicate more negative attitudes toward migration.

#### Security

Three items were selected (S1, S2, and S3) as a measure of perceived security (See Table S1 for a full list of items). These questions measured participants’ self-reported attitudes about the extent to which they were worried about their security from different threats, including a war involving their country, a terrorist attack, and a civil war. The items use a 4-point Likert-type scale ranging from “Very much” (1) to “Not at all” (4). Higher scores on the four items indicate less worry about these threats, so they indicate higher levels of perceived security.

#### National identity

Four items were selected (N1, N2, N3, and N4) as a measure of the strength of national identity (See Table S1). These questions measured participants’ self-reported pride in their country and closeness to different bodies, including their town, city, and country. The items use a 4-point Likert-type scale ranging from “Very proud” (1) to “Not proud at all” (4) and “Very proud”, and “Very close” (1) to “Not close at all” (4). Higher scores on the four items indicate a weaker sense of national identity; items were reverse scored for higher scores to reflect a stronger identity.

### Data analysis

#### Measurement validation

**Figure 3:**
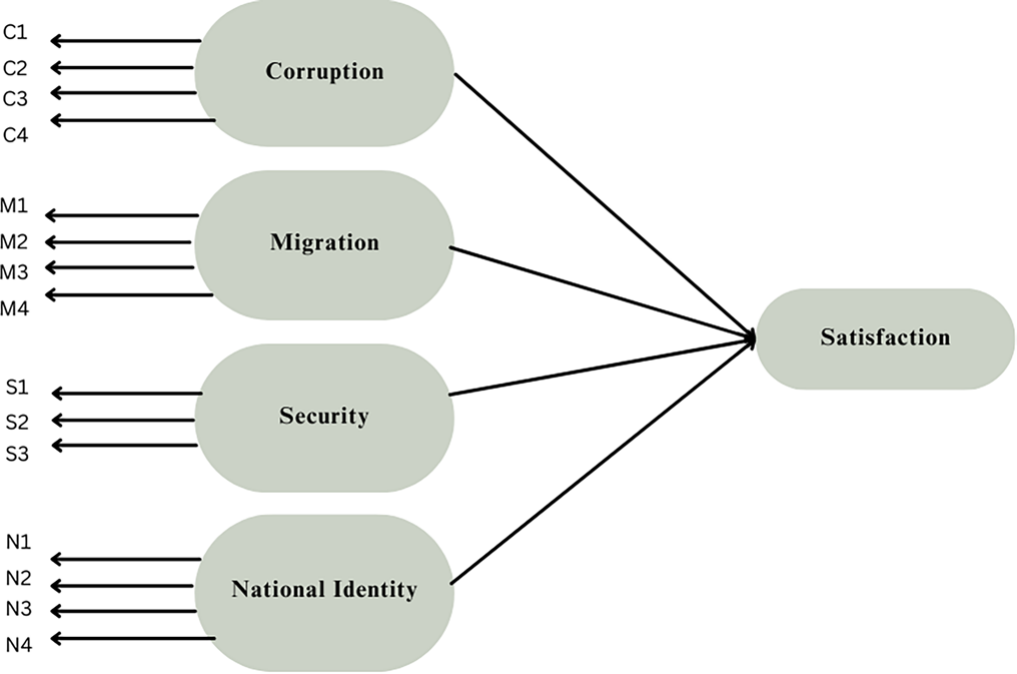
Conceptual model.

As mentioned above, we selected a specific set of questions to measure each factor. This selection was based on the classification from the World Values Survey and related literature, the average variance extracted, Cronbach's α, the estimate of the standardized path list, and the *p*-value of the unstandardized path list^11,90^. Cronbach’s α is a measure of internal consistency used to assess how well each item relates to the construct. First, we conducted a reliability analysis for each construct, removing unreliable indicators during this process. Then, we performed path analysis using confirmatory factor analysis (CFA). Insignificant indicators were iteratively removed to refine the model. We aimed to maintain an acceptable level of Cronbach’s alpha for each construct and acceptable standardized path coefficient (0.6 and above), and a significant *p*-value (less than 5%) for each indicator. The final list of indicators includes questions C1 to C4 for corruption, M1 to M4 for Migration, S1 to S3 for Security, and N1 to N4 for National Identity. The final conceptual model is illustrated in Fig. 3. Table 1 presents the reliability measures, which include the standardized correlation coefficient with total observed indicators within the same construct and Cronbach’s α. From Table 1, Cronbach’s alpha values are higher or close to the acceptable reliability value of 0.7, and the standardized correlation coefficient with the total shows a high level of association.

**Table 1:** Survey measurement validation.

Factor	Correlation with total	Cronbach’s, α	AVE	Estimate (p-value)	Standardized estimate
Corruption		0.833287	0.665		
C1	0.764506			0.65355 (< 0.0001)	0.81774
C2	0.820995			0.50760 (< 0.0001)	0.64783
C3	0.763213			0.65192 (< 0.0001)	0.82444
C4	0.805368			0.57019 (< 0.0001)	0.69648
Migration		0.738901	0.523		
M1	0.667917			0.58361 (< 0.0001)	0.67450
M2	0.686849			0.55185 (< 0.0001)	0.63638
M3	0.683055			0.53038 (< 0.0001)	0.62911
M4	0.680846			0.54506 (< 0.0001)	0.63505
Security		0.884911	0.799		
S1	0.839939			0.88703(< 0.0001)	0.84189
S2	0.804425			0.96761 (< 0.0001)	0.90644
S3	0.865450			0.88800 (< 0.0001)	0.79950
National identity		0.731415	0.540		
N1	0.324266			0.33539(< 0.0001)	0.38248
N2	0.573686			0.54303 (< 0.0001)	0.72970
N3	0.646252			0.74480 (< 0.0001)	0.75584
N4	0.568497			0.50793(< 0.0001)	0.63707

The model above has several hypotheses:

H_1_: Perceived corruption has a significant impact on individuals’ satisfaction.

H_2_: Attitudes toward migration have a significant impact on individuals’ satisfaction.

H_3_: Perceived security has a significant impact on individuals’ satisfaction.

H_4_: The strength of national identity has a significant impact on individuals’ satisfaction.

#### Average variance extracted and confirmatory factor analysis

First convergent validity assessment using the average variance extracted (AVE) values was conducted. Table 1 presents the AVE values, all of which exceed the required minimum threshold of 0.50^92^. Next, we tested discriminant validity, by comparing all the diagonal values, which are the square root of the AVEs, with the correlations between the constructs in the off-diagonal position as shown in Table 2. Overall, the results showed that the square roots of the AVEs for the constructs Corruption, Migration, Security, and National Identity are higher than the correlations of these constructs with other latent variables in the path model, which indicates that the construct measures empirically demonstrate discriminant validity^93^. Additionally, we performed a confirmatory factor analysis (CFA) to ensure that no further indicators needed to be removed. The results of the CFA, including the parameter estimates, p-values, and standardized path estimates, are reported in Table 1. All p-values are significant at the 5% error level. In addition, the standardized parameter estimate for each indicator is over 0.6. Table 3 summarizes the goodness-of-fit statistics for the CFA model. The SRMR is below 0.05, while both the GFI and BCFI are above 0.9, indicating an acceptable fit.

**Table 2:** Fornell–Larcker criterion table.

Factor	Corruption	Migration	Security	National Identity
Corruption	0.815	–	–	–
Migration	0.017	0.723	–	–
Security	− 0.055	− 0.172	0.894	–
National identity	− 0.008	0.034	− 0.071	0.735

**Table 3:** Goodness of fit for CFA.

Criteria	Value
Standardized root mean square residual (SRMR)	0.0260
Goodness of fit index (GFI)	0.9808
Bentler comparative fit index (BCFI)	0.9788

#### Structural equation modeling

To understand the impact of the exogenous factors (corruption, migration, security, and national identity) on the endogenous construct (satisfaction), we used structural equation modeling (SEM). The polychoric correlation was used in the SEM analysis to address the ordinal nature of the outcome variable. The analysis was performed using PROC CALIS in SAS software. The model’s goodness-of-fit results, shown in Table 4, indicate an acceptable fit. The path estimates, and their p-values are summarized in Table 5. According to Table 5, all indicators are significant (p-value < 0.05). For the structural relationships, satisfaction is negatively impacted by corruption and national identity, and positively impacted by security. On the other hand, migration does not have a significant impact on satisfaction with a p-value of 0.1491.

**Table 4:** Measures of fit for SEM modeling*.*

Criteria	Value
Standardized root mean square residual	0.0552
Goodness of fit index	0.9401
Bentler comparative fit index (incremental index)	0.9443

**Table 5:** SEM path list.

Path	Standardized estimate	P-value for the unstandardized estimate
Corruption → C1	0.86264	< 0.0001
Corruption → C2	0.81180	< 0.0001
Corruption → C3	0.82486	< 0.0001
Corruption → C4	0.72372	< 0.0001
Migration → M1	0.72966	< 0.0001
Migration → M2	0.58491	< 0.0001
Migration → M3	0.63178	< 0.0001
Migration → M4	0.64293	< 0.0001
Security → S1	0.83386	< 0.0001
Security → S2	0.98681	< 0.0001
Security → S3	0.77055	< 0.0001
National → N1	0.37929	< 0.0001
National → N2	0.79235	< 0.0001
National → N3	0.99082	< 0.0001
National → N4	0.81842	< 0.0001
Satisfaction → Q49	1.01156	< 0.0001
Corruption → Satisfaction	− 0.16594	< 0.0001
Security → Satisfaction	0.09954	0.0011
Migration → Satisfaction	− 0.04919	0.1491
National → Satisfaction	0.08116	0.0050

#### Multiple-group models

Conducting a multi-group analysis is used to test the path significance across different countries. For instance, countries may vary in their support for migration and levels of polarization^94^. Additionally, national identity varies in countries due to factors such as historical legacies, state-building strategies, and social policies^95^. Therefore, employing multi-group analysis is essential to comprehend these variations. To determine if the same path structure was valid, based on the country demographic, fully constrained SEM models were created for the following countries: Kenya, Armenia, Maldives, Morocco, and Venezuela. All parameters were held identically across all models. The results indicated that the path structures were consistent. The goodness-of-fit statistics for the multi-group SEM models demonstrate a good fit, as illustrated in Table 6. Moreover, the parameters for security and migration were found to be insignificant, with p-values over 0.05.

**Table 6:** Measure of fit for multi-group models—original framework.

Criteria	Value
Standardized root mean square residual	0.1832
Chi-square	2171.9193
Chi-square DF	545
Goodness of fit index	0.9320
Bentler comparative fit index (incremental index)	0.9151

Although both security and migration are individually insignificant, there is a significant correlation between these constructs across all countries, as evidenced by the covariance matrix with a p-value of (< 0.0001). Upon further investigation, including a review of the literature, we found a notable relationship in which negative attitudes toward migration influence perceived security. The association between migration attitudes and perceived security aligns with realistic group conflict theory (RGCT), as competition for resources primes negative attitudes toward out-groups^96,97^. Accordingly, negative attitudes toward out-group members, such as immigrants, lead to heightened feelings of threat and a lack of perceived security, including feelings of fear, anxiety, and stress^97,98^.

**Figure 4:**
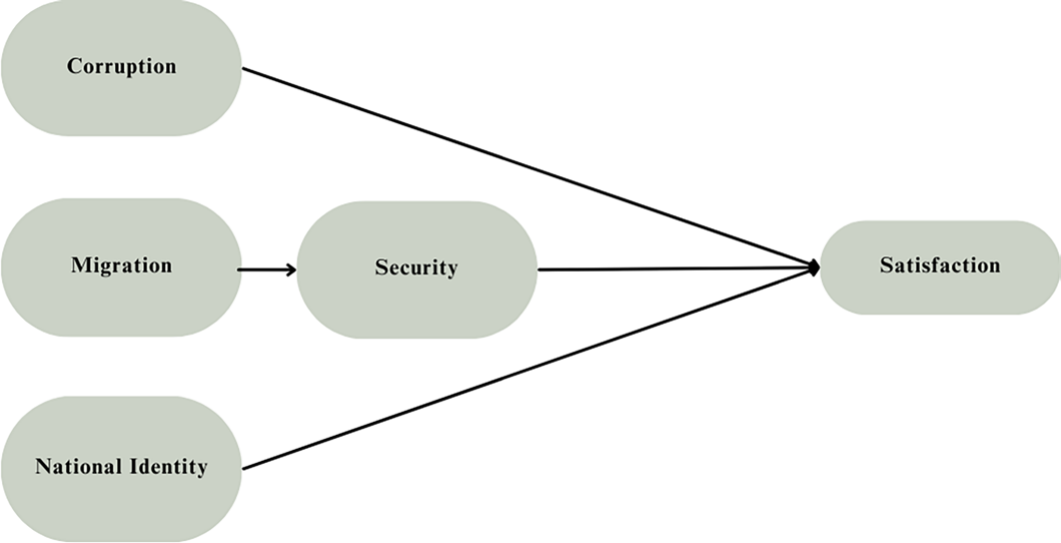
Conceptual model of the modified framework.

Consequently, we modified the framework by adding a path from migration to security, leading to a direct relationship between migration and security, as well as an indirect effect of migration on life satisfaction through security. Tables S2 and S3 show the goodness-of-fit and the SEM path list respectively. The modified framework, illustrated in Fig. 4, yielded the significance of all factors across all countries in 2021. The goodness-of-fit statistics for the multi-group modified SEM models, as shown in Table 7, demonstrate a good model fit.

**Table 7:** Measure of fit for multi-group models—modified framework.

Criteria	Value
Standardized root mean square residual	0.0855
Chi-square	1892.1533
Chi-square DF	440
Goodness of fit index	0.9338
Bentler comparative fit index (incremental index)	0.9231

##### Validation

To capture potential variations in the model across years, SEM was analyzed, but we included data from multiple years (2017 to 2021), rather than limiting it to 2021. This broader scope provides a more comprehensive understanding of the relationships between Migration, Corruption, Security, National Identity, and Satisfaction. The model’s goodness-of-fit results are shown in Table 8, indicating an acceptable fit across all years as GFI and BCFI are both higher than 0.9. The path estimates, standard errors, and their p-values are summarized in Table 8. According to Table 8, all indicators are significant (p-value < 0.05) in each year analyzed. For the structural relationships, satisfaction is negatively impacted by corruption in all years, while national identity generally has a positive impact. Security has positive impact on satisfaction while Migration consistently shows a negative impact on security across all years studied.

**Table 8:** SEM path list—modified framework.

Year	Path list	Standardized estimate	P-value for the unstandardized estimate	Goodness of fit index	Bentler comparative fit index
2017	Migration → Security	− 0.1718	< 0.0001	0.9494	0.9305
	Corruption → Satisfaction	− 0.0460	< 0.0001		
	Security → Satisfaction	0.01910	< 0.0001		
	National Identity → Satisfaction	0.1325	< 0.0001		
2018	Migration → Security	− 0.1641	< 0.0001	0.9549	0.9459
	Corruption → Satisfaction	− 0.0480	< 0.0001		
	Security → Satisfaction	0.0208	0.0002		
	National Identity → Satisfaction	0.1380	< 0.0001		
2019	Migration → Security	− 0.1625	< 0.0001	0.9467	0.9413
	Corruption → Satisfaction	− 0.0467	< 0.0001		
	Security → Satisfaction	0.0205	0.0002		
	National Identity → Satisfaction	0.1345	< 0.0001		
2020	Migration → Security	− 0.1664	< 0.0001	0.9617	0.9644
	Corruption → Satisfaction	− 0.0417	< 0.0001		
	Security → Satisfaction	0.0179	0.0002		
	National Identity → Satisfaction	0.1199	< 0.0001		
2021	Migration → Security	− 0.2031	< 0.0001	0.9750	0.9668
	Corruption → Satisfaction	− 0.0562	< 0.0001		
	Security → Satisfaction	0.0197	0.0002		
	National Identity → Satisfaction	0.1618	< 0.0001		

The countries participating each year varied. In 2017, the respondents were from Greece (7%), Serbia (14%), Argentina (5%), Bolivia (19%), Russia (10%), and the USA (45%). In 2018, the respondents came from Andorra (4%), Australia (6%), Bangladesh (2%), Brazil (2%), Chile (1%), Colombia (7%), Germany (4%), Ecuador (4%), Hong Kong (8%), Indonesia (10%), Iraq (3%), Kazakhstan (2%), South Korea (7%), Lebanon (3%), Mexico (6%), Malaysia (7%), Nigeria (4%), Pakistan (4%), Peru (4%), Puerto Rico (4%), Romania (1%), Thailand (4%), and Turkey (3%). In 2019, the respondents were from Cyprus (4%), Japan (3%), Macao (18%), Philippines (32%), Tunisia (16%), and Taiwan (27%). In 2020, the respondents included Canada (42%), Ethiopia (4%), Guatemala (9%), Iran (4%), Kyrgyzstan (4%), Mongolia (17%), Nicaragua (8%), New Zealand (2%), Ukraine (1%), and Zimbabwe (9%). Finally, in 2021, the respondents were from Armenia (8%), Kenya (20%), Morocco (31%), Maldives (10%), and Venezuela (31%).

#### Ordinal logistic regression analysis (OLR)

To determine how different demographics predict life satisfaction, we developed an ordinal logistic regression model, focusing on 2021 data. In this model, we treat the satisfaction variable from the survey data as ordinal rather than continuous due to its representation of ordered categories. The scale from 1 to 10 reflects different levels of satisfaction, but the numerical differences between the ratings may not consistently denote equal increments in satisfaction, making it more appropriate to consider it as an ordinal scale for statistical analysis.

The model included several demographic factors, such as country, gender, relationship status, social class, and religion. The countries involved in the study were Armenia, Kenya, Venezuela, Morocco, and the Maldives. To simplify the analysis, relationship status and religion were recoded as binary variables, indicating whether the respondent was in a relationship or not, and whether they identified as religious or not. Table 9 below presents the Type 3 analysis of effects results of the ordinal regression, which explains the statistical significance of each demographic while controlling for the effect of the others. Interestingly, gender was not statistically significant (p-values > 0.05), while country, relationship status, social class, and religion were significant (p-values < 0.05) predictors of life satisfaction.

**Table 9:** OLR type 3 analysis of effects.

Effect	P-value
Country	< 0.0001
Gender	0.7790
Relationship	0.0009
Social class	0.0123
Religion	0.0034

The ordinal logistic regression analysis provides insights into the associations between the response variable and various predictor variables using the Odds Ratios. Table 10 presents the odds ratios for the significant variables. As shown in Table 10 below, all countries seem to have a higher satisfaction compared to Kenya. When comparing individuals in a relationship to those who are not, the odds of satisfaction are 22.7% higher for individuals in a relationship. As for socioeconomic class, the odds of satisfaction for the lower middle class are 32.8% higher compared to individuals in the lower class. In addition, individuals in the upper class and upper middle class have odds that are 46.4% and 45.5% greater, respectively, than those in the lower class. Moreover, individuals who identified as religious have 42.8% higher odds of satisfaction than individuals who identified as not religious.

**Table 10:** Odds ratios for the significant variables.

Effect	Point estimate
Armenia vs Kenya	2.215
Morocco vs Kenya	1.721
Maldives vs Kenya	2.245
Venezuela vs Kenya	2.452
In relationship vs no relationship	1.227
Lower middle vs lower	1.328
Upper vs lower	1.464
Upper middle vs lower	1.455
Working vs lower	1.215
Religious vs not religious	1.428

## Discussion and conclusion

In this paper, we explored how different socio-political factors contribute to individual life satisfaction. We developed a conceptual model, as shown in Fig. 3, illustrating how corruption, migration, security, and national identity influence life satisfaction. Through reliability analysis, AVE, and CFA, we selected relevant items in the WVS to measure each factor. Then, using these items, we constructed an SEM to evaluate the effect of each socio-political dimension on life satisfaction. We found that perceived corruption, perceived security, and national identity were significant dimensions that impact life satisfaction.

Consistent with previous research, our findings supported hypothesis H_1_. The negative relationship between perceived corruption and life satisfaction aligns with the work of Helliwell^25^, who found that individuals perceiving more corruption report lower levels of life satisfaction and subjective well-being compared to those perceiving less corruption. Since perceived corruption relies on the regular interactions between individuals and their surrounding establishments, it is unsurprising that the many negative outcomes of corruption extend to life satisfaction^99^.

Furthermore, results from our SEM model support H_3_, indicating that perceived security has a positive impact on life satisfaction. However, this finding did not align with Sortheix and Lönnqvist^100^, who found that high security values were associated with lower life satisfaction across 25 European nations. One possible explanation for such results is that high security values reflect a greater need to protect oneself and are more likely to be connected to feelings of threat than security^101^. Our findings also support H_4_, as the strength of national identity had a significant impact on individuals’ life satisfaction. The positive association in our analyses aligns with the literature on national identity, and its enhancement of satisfaction with life through providing a sense of connection and belonging^43,48^.

The current findings of our SEM analysis did not support H_2_, implying that negative attitudes toward migration have no direct effect on an individual's satisfaction. Later multi-group analyses examining the impact of country on the validity of our conceptual model indicated an indirect effect of attitudes toward migration on life satisfaction through perceived security. The association between negative attitudes toward migration and perceived security, which was negative, aligns with realistic group conflict theory (RGCT), in which negative attitudes toward outgroups such as immigrants cause heightened perceptions of threat and a lack of security, including feelings of fear and intergroup anxiety^96,98^.

We extended our modified framework, including the indirect effect of attitudes toward migration on life satisfaction, to other years in Wave 7 of the WVS, testing our framework across 42 countries. Findings of the modified framework indicate significance across all four socio-political factors, supporting the conceptual model in Fig. 4. We conclude that perceived corruption, perceived security, and national identity have a direct significant impact on life satisfaction, while attitudes toward migration have an indirect negative effect on life satisfaction through perceived security.

Furthermore, we conducted OLR to better understand the relationship between demographic factors and life satisfaction. As seen in Table 9, we found that gender was not a significant factor, which supports the recent research challenging gender differences in outcomes such as life satisfaction^70^. Given the lack of consensus about sex differences in life satisfaction in the literature, our findings contribute to the literature by determining that no sex differences in life satisfaction were found in the examined ecological context.

Our findings also revealed that relationship status was a significant predictor of life satisfaction, aligning with the literature review, which indicates that being in a relationship is significantly associated with higher levels of life satisfaction. Single individuals, as shown in the study by Adamczyk & Segrin^55^, tend to have lower levels of life satisfaction and social support, and higher levels of loneliness compared to individuals in relationships. Furthermore, OLR analysis indicates that individuals in a relationship have higher odds of satisfaction than those not in a relationship.

The analysis also revealed the significance of social class, which is not surprising given the literature that links socioeconomic status with life satisfaction by meeting people's needs, as noted by Gitmez and Morcöl^60^. Our results further support the relationship between socioeconomic status and life satisfaction, showing that individuals in the Lower Middle class, Upper class, and Upper Middle class had higher levels of satisfaction compared to those in the Lower class. This aligns with previous research indicating a positive relationship between socioeconomic status and life satisfaction. For example, studies have shown that individuals from more affluent families report higher levels of life satisfaction compared to those from less affluent families^62^.

Meanwhile, the OLR model revealed that religious affiliation was significant, which can be supported by research, like Hayward et al.’s^76^, which stated that individuals who have religious affiliations tend to have enhanced psychological well-being, social support, and health behaviors compared to those who identify as atheists or agnostics. Our findings support this association further since they indicate that religious people have greater odds of life satisfaction than non-religious people. This is consistent with earlier studies that showed a link between religion and life satisfaction. For instance, research has shown that social religiosity encourages the growth of social networks and social support, which in turn indirectly increases life happiness^75^. Therefore, current findings offer additional evidence in favor of the positive impacts of religious connection on life happiness.

Lastly, our analysis revealed that country was significant, which aligns with cross-national research providing evidence that national indicators such as national wealth, environmental conditions, and human development are important when considering life satisfaction in a regional or national context^81^. Kenya’s reported level of life satisfaction in our analysis, which is the lowest among the studied countries, supports Degutis et al.’s^88^ research on the positive relationship between GDP and life satisfaction, as Kenya has the lowest GDP per capita among countries in 2021.

Overall, our findings make important contributions to the existing literature by presenting a conceptual model that examines the relationship between life satisfaction and multiple socio-political factors of perceived corruption, perceived security, attitudes toward migration, and national identity. Additionally, findings develop the current understanding of the impact of demographic variables that are subjects of debate in the literature, such as sex, relationship status, social class, and religious denomination on life satisfaction. Specifically, we determine how these individual-level variables can shape life satisfaction within the ecological context of the present study.

This study can advance the current understanding of life satisfaction from a socio-political perspective and can potentially be used to enhance life satisfaction in a population through implementing policies that consider satisfaction and promoting a local culture of positivity and happiness among community members^102^. Enhancing life satisfaction at the micro level using the presented conceptual model can be of particular importance to governing authorities such as the Ministry of Happiness in the United Arab Emirates, whose main objectives include promoting happiness, well-being, and development^102^. Importantly, while national initiatives worldwide focus on using happiness to inform policies, integrating a cognitive model of life satisfaction can be equally important for evaluating overall success and well-being^103^.

Limitations of the current study include the influence of demographic factors, other than country, on shaping life satisfaction and moderating the effects observed in the modified framework. However, our multi-group models were limited to country, as existing research supported the relevant impact of country on the studied factors^94,95^. As a recommendation for future research, it is important to consider how culture interacts with socio-political factors, influencing social and political spheres. Future research could investigate whether countries can be clustered based on their life satisfaction and their position on the collectivism-individualism scale.

### Supplementary Information

Supplementary Tables.

## Data Availability

The data set used in this research is available at https://www.worldvaluessurvey.org/WVSDocumentationWV7.jsp.
