# Supplementary material for: Statistical analysis of the effect of socio-political factors on individual life satisfaction
Source: Sci Rep. 2024 Aug 24;14:19677. doi: 10.1038/s41598-024-70067-5 (PMC11344825; doi:10.1038/s41598-024-70067-5)
Supplement: Supplementary file 1 — Supplementary Tables. [file 41598_2024_70067_MOESM1_ESM.docx]

# Supplementary

**Table S1** Items on the WVS, corresponding coded items, and the question for each item

| Measure | Items on WVS | Coded Items | Question |
| --- | --- | --- | --- |
| Life Satisfaction | Q49 | Satisfaction | All things considered, how satisfied are you with your life as a whole these days? |
| Corruption | Q113 | C1 | Among state authorities, how many do you believe are involved in corruption? |
|  | Q114 | C2 | Among business executives, how many do you believe are involved in corruption? |
|  | Q115 | C3 | Among local authorities, how many do you believe are involved in corruption? |
|  | Q116 | C4 | Among civil service providers (police, judiciary, civil servants, doctors, teachers), how many do you believe are involved in corruption? |
| Migration | Q124 | M1 | For each  of the following statements about the effects of immigration, please, tell me whether you agree or disagree with it: Increases the crime rate. |
|  | Q126 | M2 | For each  of the following statements about the effects of immigration, please, tell me whether you agree or disagree with it: Increases the risks of terrorism |
|  | Q128 | M3 | For each  of the following statements about the effects of immigration, please, tell me whether you agree or disagree with it: Increases unemployment |
|  | Q129 | M4 | For each  of the following statements about the effects of immigration, please, tell me whether you agree or disagree with it: Leads to social conflict |
| Security | Q146 | S1 | To what degree are you worried about a war involving your country? |
|  | Q147 | S2 | To what degree are you worried about a terrorist attack? |
|  | Q148 | S3 | To what degree are you worried about a civil war? |
| National Identity | Q254 | N1 | How proud are you to be [country’s nationality]? |
|  | Q255 | N2 | How close do you feel to your [village, town or city]? |
|  | Q256 | N3 | How close do you feel to your [county, region, district]? |
|  | Q257 | N4 | How close do you feel to your [Country]? |

**Table S2** Measures of Fit for SEM Modeling - Modified Framework

| Criteria | Value |
| --- | --- |
| Standardized Root Mean Square Residual | 0.0544 |
| Goodness of Fit Index | 0.9761 |
| Bentler Comparative Fit Index (Incremental Index) | 0.9653 |

**Table S3** SEM Path List - Modified Framework

| Path | Standardized Estimate | Standard Error |  | Unstandardized P-value |
| --- | --- | --- | --- | --- |
| Corruption → C1 | 0.81453 | 0.01225 |  | <.0001 |
| Corruption → C2 | 0.64669 | 0.01281 |  | <.0001 |
| Corruption → C3 | 0.82211 | 0.01207 |  | <.0001 |
| Corruption → C4 | 0.69444 | 0.01318 |  | <.0001 |
| Migration → M1 | 0.67275 | 0.01567 |  | <.0001 |
| Migration → M2 | 0.64077 | 0.01575 |  | <.0001 |
| Migration → M3 | 0.62522 | 0.01537 |  | <.0001 |
| Migration → M4 | 0.63815 | 0.01557 |  | <.0001 |
| Security → S1 | 0.83775 | 0.01210 |  | <.0001 |
| Security → S2 | 0.90971 | 0.01124 |  | <.0001 |
| Security → S3 | 0.79827 | 0.01314 |  | <.0001 |
| National → N1 | 0.34923 | 0.01298 |  | <.0001 |
| National →N2 | 0.71180 | 0.01061 |  | <.0001 |
| National → N3 | 0.87036 | 0.01241 |  | <.0001 |
| National → N4 | 0.68073 | 0.01312 |  | <.0001 |
| Satisfaction → Q49 | 0.99653 | 0.01182 |  | <.0001 |
| Migration → Security | -0.20642 | 0.01838 |  | <.0001 |
| Corruption → Satisfaction | -0.09360 | 0.04575 |  | <.0001 |
| Security → Satisfaction | 0.05778 | 0.04976 |  | 0.0012 |
| National→ Satisfaction | 0.10953 | 0.04619 |  | <.0001 |
